# 

**Published:** 1967-07

**Authors:** 


### Contents

| | | Page |
|---|---|---|
| WILLIAM BUDD ........................................ | *R. C. Wofinden* | 63 |
| THE NATURE AND THE MODE OF PROPAGATION OF PHTHISIS ........................................ | *William Budd* | 68 |
| LOUIS NAPOLEON AND HIS DOCTORS ... | *S. F. Marwood* | 71 |
| CASE REPORTS .............................. | *A. A. J. Barros D'Sa* | 83 |
| JENNER .................................................................. | | 95 |

